# Can Preterm Labour Be Predicted in Low Risk Pregnancies? Role of Clinical, Sonographic, and Biochemical Markers

**DOI:** 10.1155/2014/623269

**Published:** 2014-10-22

**Authors:** Reva Tripathi, Shakun Tyagi, Nilanchali Singh, Yedla Manikya Mala, Chanchal Singh, Preena Bhalla, Siddhartha Ramji

**Affiliations:** ^1^Department of Obstetrics and Gynaecology, Maulana Azad Medical College, New Delhi 110002, India; ^2^Department of Microbiology, Maulana Azad Medical College, . New Delhi, India; ^3^Department of Paediatrics, Maulana Azad Medical College, . New Delhi, India

## Abstract

*Background and Objectives.* This is a prospective nested cohort study conducted over a period of 3 years. 2644 women were recruited, out of which final analysis was done for 1884 women.* Methods*. Cervicovaginal and blood samples were collected for all recruited women. Out of these, 137 women who delivered before 35 weeks were treated as cases and equal number of matched controls were chosen. Analysis of samples for serum G-CSF, AFP, ferritin, and cervicovaginal interleukin-6 and IGFBP-1 was done.* Results*. Poor orodental hygiene, which can be a social marker, was significantly more common in women who delivered preterm (*P* = 0.008). Serum alkaline phosphatase and serum ferritin were found to be significantly associated with preterm deliveries. The 90th percentile value of these parameters was considered as cut-off as there is no specific cut-off.* Conclusions*. Our study did not prove usefulness of any predictive marker. Serum ferritin and alkaline phosphatase were found to have correlation but their values are affected in many conditions and need to be elucidated with caution. Larger studies are needed for predicting preterm labour in asymptomatic women.

## 1. Introduction

Prematurity continues to be the major cause of neonatal morbidity and mortality across the world accounting for an enormous 70% of neonatal deaths in nonanomalous babies [1]. Despite decades of research, we are no closer to finding answers in terms of prediction and hence prevention of preterm labour. This is in sharp contrast to the increasing rates of preterm births across the world. This increase is in part attributable to increase in artificial reproductive techniques and multiple pregnancies. Efforts to predict preterm delivery have classically been based on history and examination findings only. But these risk assessment scores have poor predictive values [2, 3]. Some studies have found cervical length as measured by transvaginal ultrasound and cervicovaginal IGFBP-1 as most strongly and consistently associated with subsequent spontaneous preterm birth [4]. Increased level of cervicovaginal interleukin-6 (IL-6) at 24 weeks has also been associated with preterm delivery [5]. New biochemical markers like increased levels of serum ferritin, serum granulocyte colony-stimulating factor, serum alkaline phosphatase, and serum alpha-fetoprotein have also been associated with increased incidence of preterm delivery [6, 7]. Combining these markers with clinical findings and ultrasonography has also contributed significantly to increased predictive values [4]. We wanted to validate various screening tests for predicting preterm labour in low risk women as there is a known effective intervention (i.e., progesterone) for its prevention. This study was undertaken to evaluate the role of biomarkers and cervical length in prediction of preterm labour in asymptomatic low risk women in an Indian population.

## 2. Materials and Methods

This prospective study was conducted at a tertiary care teaching hospital over a period of 3 years. All low risk women with singleton pregnancy who visited the antenatal clinic of a single unit between 24 and 27 weeks and 6 days were consecutively recruited after counseling and an informed consent. Women either with certain last menstrual periods or having a first trimester ultrasound were recruited in study. The study was approved by the ethical committee of the hospital.

Considering the rate of preterm delivery at 35 weeks being approximately 5% at our centre, for a 95% confidence level and 80% power for majority of factors to be studied, 2600 pregnant women needed to be studied. Only low risk women were included in the study. Women with history suggestive of cervical incompetence, previous preterm delivery (defined as less than 37 weeks) or previous cervical surgery, preexisting medical disorders like chronic hypertension, diabetes mellitus, heart disease, and SLE, and so forth, were excluded from the study. Women with major degree placenta previa or gross congenital anomaly in the foetus were also excluded. All women underwent routine antenatal care with thorough history taking and obstetric examination.

A 10 mL venous blood sample was collected in a vacutainer from all the women recruited in study and transported within three hours to be stored at −70° Celsius for assessment of the following biomarkers: serum ALP, AFP, ferritin, G-CSF, and interleukin-6. Sterile speculum examination was done and cervical swab taken for smear examination after gram staining. Nugent score was calculated. pH of cervical secretion was determined using litmus paper and a pH value of more than 4.5 was taken as “abnormal.” Cervicovaginal secretion was collected for assessment of insulin like growth factor binding protein (IGFBP-1) and interleukin-6.

For this study a nested cohort was used; that is, samples of all recruited patients were collected and stored. A smear was made on slide using swab and air-dried to fix it. Two dacron swabs were soaked in cervical secretions by placing them at the external os for 15 seconds to absorb cervical secretions and stored in a buffer solution containing sodium phosphate, sodium chloride, EDTA, Tween-20, bovine serum albumin, aprotinin, and proclin 300. These were then stored in deep freeze (−70° Celsius) to be tested later for IGFBP-1 and interleukin-6.

Those who delivered at less than 35 weeks gestation were treated as* cases*. An equal number of women matched with respect to parity and age amongst the remaining recruited women delivering at more than 37 weeks were treated as* controls*. Using efficient transport and storage of samples, only samples of cases and controls were analyzed, to reduce the financial burden of kits required for biochemical analysis. Analysis of the samples was done for patients who eventually delivered preterm and their matched controls. Analysis of samples for serum granulocyte colony-stimulating factor, serum alpha-fetoprotein, serum ferritin, and cervicovaginal interleukin-6 was done in microbiology department of the institution using ELISA kits for these parameters. For testing IGFBP-1, the swabs were placed into the specimen extraction solution provided with the kit (*Rapid Actim Partus test*,* Medix Biochemica, Kauniainen, Finland*) and swirled around vigorously for 10 seconds. The swab was then withdrawn and the dipstick provided in the kit was placed in the specimen extraction solution and held there till the liquid front reaches the result area. The dipstick was then withdrawn and results were interpreted after 5 minutes. The appearance of two blue lines was taken as positive whereas only one blue line at the end of five minutes was taken as negative. Tests for cervicovaginal IGFBP-1 and IL-6 were standardized prior to analyses. Serum levels of iron and alkaline phosphatase were tested.

A transvaginal scan was done in the same sitting. All the scans were performed by the same sonographer using 5 MHz to 9 MHz transvaginal transducer of HD 11 Philips ultrasound machine and cervical length was measured as recommended. A cervical length of less than 25 mm was defined as “short” cervix. The treating obstetricians were blinded to the results of these tests. All women were followed till delivery and obstetric and neonatal outcomes noted. The primary outcome was preterm delivery defined as delivery before 37 weeks. Women who required iatrogenic preterm delivery were excluded from the final analysis. Serum AFP, ALP, IL-6, and GCSF and serum ferritin were measured in the stored samples of women who had spontaneous preterm delivery before 37 weeks and an equal number of matched controls. Since there are no defined cut-offs for these biomarkers, values of more than 90th percentile were taken as high. Serum ferritin less than the 25th percentile was taken as “low.”

### 2.1. Statistical Analysis

SPSS Version 17 and Microsoft Excel 2007 have been used for statistical analyses. *χ* ^2^-test has been used for comparison of categorical variables and Student's *t*-test has been used for comparison of continuous variables. *P* values < 0.05 have been considered as significant. Odds ratios have been computed subsequently.

## 3. Results

A total of 2644 women were recruited for the study. 532 women required iatrogenic preterm delivery prior to 37 weeks and were excluded. One woman underwent cerclage at 24 weeks and was excluded from the analysis. There were 3 maternal mortalities which were excluded. 224 (8.4%) women were lost to follow-up. Thus the final analysis was done for 1884 women. Demographic data for the study population is summarized in Table 1. Most of the demographic characteristics were similar in both the groups except family type and orodental hygiene. Although mental stress was not studied separately; family type was studied in an attempt to analyse whether this could be a surrogate indicator of mental stress which in turn is associated with preterm deliveries. Extended family staying together with a young couple is a feature typical of Indian culture. In our study joint family setup was significantly associated with preterm delivery (*P* = 0.042). This might be explained by the increased physical and mental stress associated with larger extended families. Good orodental hygiene was significantly more common in case of women who delivered at term as compared to women who delivered preterm (*P* = 0.008). The association of poor orodental hygiene with preterm birth needs further elucidation in the Indian scenario.

The various predictive markers in both the cases and control group are shown in Table 2. Only serum alkaline phosphatase and serum ferritin were found to be significantly associated with preterm deliveries. The serum alkaline phosphatase which was studied in this study was not placental-specific and hence could have been raised due to many other reasons. The serum ferritin could be raised in anaemia, which is so prevalent in Indian women; hence, serum iron was done in these women. Hence, before taking into account the interpretation of these markers, other possible pathologies should be ruled out. The difference in the level of serum iron in the preterm and control group was not statistically significantly.

The 90th percentile value of biochemical laboratory parameters was considered as cut-off when deciding relevance as there is no specific cut-off for these markers. The distribution of the various biochemical laboratory parameters with respect to 90th percentile value in the cases and controls has therefore also been depicted in Table 3. Serum alkaline phosphatase (>90th percentile) was significantly more common in preterm group with high odds ratio of 3.0315. No significant association was seen in other markers.  Table 4 shows sensitivity, specificity, and positive and negative likelihood ratios (LR) of some of the markers.

## 4. Discussion

Prematurity is a leading cause of neonatal and infant morbidity and mortality and many times it occurs unexpectedly in low risk women. Many biochemical and imaging predictors have been evaluated as screening test of preterm labour in low risk women. An ideal screening test should have a high sensitivity and specificity and should be widely available, easy to perform, reproducible, and accurate. More importantly, an effective intervention should be available to ameliorate the condition for which the screening test is positive. With a solid body of evidence establishing the role of vaginal progesterone in prevention of preterm labour, the search for predictors of spontaneous preterm labour has intensified [8, 9]. Though many biochemical markers have been evaluated, sonographic cervical length, IGFBP-1, and fetal fibronectin in cervicovaginal secretions are most widely used in preterm labour prediction [10]. We wanted to evaluate their success in predicting it in low risk women.

The pathogenesis of preterm labour is not well understood but multifactorial etiology has been postulated. A significant amount of evidence suggests that preterm labour is mediated via infection and inflammation [11]. From study of placentae of preterm deliveries it was found that histological chorioamnionitis was more common in preterm than term deliveries [12]. Therefore, if presence of subclinical infection can be detected between 24 and 28 weeks by various biochemical markers of infection, it would be a step forward towards predicting preterm delivery. This study was planned on this premise. Various clinical, ultrasonographic, and biochemical parameters were studied.

In this study, the cases and controls were matched with respect to age, parity, and socioeconomic status; hence, these parameters were not studied. However, previous studies have reported increased risk of preterm delivery if the maternal age is less than 18 years and more than 35 years [13]. Previous studies have shown association between history of second trimester abortions and preterm delivery; no such association was observed in our study [14, 15]. The association of poor orodental hygiene with preterm birth is a relatively new area of study and though proven to have some association with preterm delivery needs further elucidation in the Indian scenario.

Cervical length less than or equal to 22–25 mm and presence of funneling have been suggested as an important marker for prediction of preterm labour and delivery [16–18]. In a systematic review, it was found that shorter the cervical length, the higher the positive likelihood ratio for preterm delivery [19]. The most common cervical length cut-off used was <25 mm. Combination with other markers like IGFBP-1 improves the predictive value [20]. Various other studies have concluded that sonographic cervical assessment may be useful in the prediction of preterm delivery, but it should be considered in association with the previous history of preterm delivery rather than in isolation [20]. However in our study cervical length and funneling were not significantly associated with preterm delivery (*P* = 0.212) refuting their efficacy in low risk women.

Various studies show that asymptomatic patients with increased serum IL-6 values between 24 and 36 weeks of gestation are at high risk for preterm delivery [21, 22]. However in our study there was no statistical correlation between levels of IL-6 in serum and preterm delivery (*P* = 0.4205). Granulocyte colony-stimulating factor is elevated in the amniotic fluid and plasma of women with chorioamnionitis and active preterm labour. A study evaluated the association of plasma granulocyte colony-stimulating factor and subsequent spontaneous preterm birth in asymptomatic pregnant women [23]. It reported that compared to term controls, increased values of granulocyte colony-stimulating factor tested at 24 weeks of gestation were found in women delivering before 28 weeks. In our study no such association was found (*P* = 0.472).

Alkaline phosphatase and alpha-fetoprotein have also been found to be significantly elevated in pregnancies associated with spontaneous preterm birth. In preterm prediction study, Moawad et al. reported association of alkaline phosphatase and alpha-fetoprotein levels with preterm birth. When alkaline phosphatase levels at 24 weeks were studied, the odds ratio for spontaneous preterm birth at <32 weeks was 6.8 (1.4–32.8) and at <35 weeks was 5.1 (1.7–15.6) [24]. In our study there was significant correlation between preterm birth and serum alkaline phosphatase levels at 24 to 28 weeks (*P* = 0.009). In the same study, increased serum alpha-fetoprotein levels at 24 weeks were associated with spontaneous preterm birth at <32 weeks (OR-8.3) and <35 weeks (OR-3.5). There was no statistically significant correlation between preterm birth and serum alpha-fetoprotein levels in our study.

Some prior studies have analysed association between serum ferritin and preterm delivery. One of them reported that, after adjusting for various possible confounding factors, the odds ratio for extreme quartiles (>64.5 versus <26.0 ng/mL) of ferritin was 1.3 (95% CI 0.8, 2.1). Stratified analyses indicated that elevated maternal serum ferritin was associated with an increased risk of preterm premature rupture of membranes (OR = 2.1; 95% CI 1.1, 4.1), but not with spontaneous preterm labour (OR = 0.9; 95% CI 0.4, 1.7) or induced preterm delivery (OR = 1.1; 95% CI 0.6, 2.0) [25]. Considering 60% prevalence rate of anaemia during pregnancy in India, serum iron levels were also done to eliminate anaemia as the confounding factor. In our study both serum ferritin and serum iron levels were less in women who delivered preterm as compared to the women who delivered at term but the difference was not statistically significant (*P* = 0.053). This reflects that in developing countries like India the etiology of preterm birth might be more related to nutrition and specifically deficiency of micronutrients like iron. Further studies are indicated.

Insulin-like growth factor binding protein-1 (IGFBP-1) is mainly secreted from fetal and adult liver. It leaks into cervical secretions when fetal membranes detach from decidua. Studies have shown that a bedside rapid strip test (Actim Partus Test) for detection of IGFBP-1 in cervical secretions provides an additional diagnostic tool for the assessment of patients presenting with preterm labour [26, 27]. In another study in which cervicovaginal sample was collected at 22-23 weeks, IGFBP-1 > 6.4 *µ*g/L had a likelihood ratio of +1.8 (95% CI 0.7–2.9) [10]. However in our study group comprised of only low risk women, IGFBP-1 levels did not significantly correlate with preterm delivery. This parameter is possibly more relevant in those patients who have already gone into preterm labour process. We also evaluated IL-6 levels in cervicovaginal secretions for predicting preterm birth; however, no significant correlation was observed. Another study evaluated cervical IL-6 levels in patients who delivered at less than 32 and 35 weeks and found it to be significantly higher in preterm deliveries as compared to their matched controls [28].

Some newer markers have been evaluated in various studies like vitamin D-binding protein, triggering receptor expressed on myeloid cells-1* *(sTREM-1), matrix metalloproteinases-* *(MMP-) 9, MMP-3, tissue inhibitor of metalloproteinases-* *(TIMP-) 1, TIMP-2, TIMP-3, and TIMP-4, and a panel of various* *cytokines, chemokines, and growth factors. But they all are still in experimental phase [29, 30].

The lack of association between serum and cervicovaginal makers of inflammation with preterm labour in low risk women points towards some etiology other than inflammation or infection for the occurrence of preterm delivery. Considering 60% prevalence rate of anaemia during pregnancy in India, serum iron levels were also done to eliminate anaemia as the confounding factor. In our study both serum ferritin and serum iron levels were less in women who delivered preterm as compared to the women who delivered at term (*P* = 0.053) but the difference was not statistically significant. This reflects that in developing countries like India the etiology of preterm labour birth might be more related to nutrition and specifically deficiency of micronutrients like Iron.

The strengths of this study were its study design, strict inclusion criteria pertaining to low risk women with no prior history of preterm delivery, and blinding of results to treating physicians. On the other hand, the drawback was smaller samples of preterm cases and controls. Since preterm labour is multifactorial, several factors may have been unaccounted for and further studies are indicated in this important arena.

## 5. Conclusion

Our study did not prove usefulness of predictive markers like serum AFP, G-CSF, and interleukin-6 and cervicovaginal insulin like growth factor binding protein (IGFBP-1) and interleukin-6. Serum ferritin and alkaline phosphatase were found to have correlation but value of the former is affected in conditions like anaemia and the latter in many other conditions like hemolysis, liver disorders, and so forth. Hence, these markers cannot be relied upon in predicting preterm labour in asymptomatic women. Larger studies evaluating these biomarkers are needed for predicting preterm labour in asymptomatic women. Role of newer markers including genetic markers and micronutrient deficiency needs elucidation.

## Figures and Tables

**Table 1:** Population characteristics in both the groups.

Characteristics	Preterm	Term	*P* value
	*n* = 137 (%)	*n* = 138 (%)	
B.M.I (mean ± SD)	22.858 (±4.147)	23.483 (±3.908)	0.1992
Literacy			
Illiterate	33 (24.09)	22 (15.94)	
Primary	22 (16.06)	18 (13.04)	
Secondary	70 (51.09)	78 (56.52)	0.172
Graduate and above	12 (8.76)	20 (14.49)	
Family type			
Joint	82 (59.85)	99 (71.74)	*0.042 *
Nuclear	55 (40.15)	39 (28.26)	
Tobacco use			
Present	1 (0.73)	1 (0.72)	1.000
Absent	136 (99.27)	137 (99.28)	
Profession			
Home maker	133 (97.08)	135 (97.83)	
Working	4 (2.92)	3 (2.17)	0.723
Type of work			
Heavy	2 (1.46)	0 (00)	
Moderate	135 (98.54)	138 (100)	0.247
Sedentary	00 (00)	00 (00)	
Domestic violence			
Present	3 (2.19)	1 (0.72)	
Absent	134 (97.81)	137 (99.28)	0.370
Parity			
0	56 (40.88%)	59 (42.75%)	
1	57 (41.61%)	55 (39.86%)	0.966
2	21 (15.33%)	22 (15.94%)	
3	3 (2.19%)	2 (1.45%)	
Abortions			
0	107 (78.10%)	102 (73.91%)	
1	24 (17.52%)	30 (21.74%)	0.875
2	4 (2.92%)	4 (2.90%)	
3	2 (1.46%)	2 (1.45%)	
Poor orodental hygiene	10 (7.30%)	14 (10.14%)	*0.008 *

**Table 2:** Various predictive markers in both the groups.

	Preterm	Term	*P* value
	(*n* = 137)	(*n* = 138)	
Nugent's score ≥8	15	13	0.725 (NS)
Sonographic			
Cervical length (cm)	3.424 (±0.6582)	3.535 (±0.627)	0.2120 (NS)
Funneling	3	1	
Length of funnel (cm)	1.475 (±0.459)	1.72 (±0)	
Diameter of os (cm)	0.25 (0.15–1.08)	0.71 (±0)	
S. interleukin-6 (pg/ml)	0 (0–100)	0 (0–100)	0.4205 (NS)
S. alpha-fetoprotein (ng/ml)	68 (0–208)	62 (0–280)	0.5462 (NS)
S. alkaline phosphatase (U/I)	212.511 (±98.538)	172.835 (±79.214)	**0.0003 (Sig)**
S. ferritin (ng/ml)	10 (2–90)	15 (1–98)	**0.0134 (sig)**
S. iron (*μ*g/dl)	54.5 (17–211)	60 (16–424)	0.2741 (NS)
S. granulocyte colony-stimulating factor (pg/ml)	10 (0–360)	15 (0–560)	0.4728 (NS)
Insulin growth factor binding protein-1			
(in cervicovaginal secretion)			
Negative	24 (88.89)	46 (88.46)	1.000 (NS)
Positive	3 (11.11)	6 (11.54)	
Interleukin-6 (pg/ml)	0 (0–2)	0 (0–2)	0.312 (NS)
(in cervicovaginal secretion)			

**Table 3:** Showing number of patients with more than 90th percentile of various predictive markers in both the groups.

	Preterm	Term	*P* value	Odds ratio
	(*n* = 137)	(*n* = 138)		(95% CI)
Serum IL-6 (>90th percentile)	14	16	0.42	0.662 (0.3053 to 1.4354)
Serum ferritin (>90th percentile)	10	20	0.05	0.464 (0.2089 to 1.0333)
Serum G-CSF (>90th percentile)	16	21	0.36	0.9205 (0.4505 to 1.8807)
Serum ALP (>90th percentile)	20	7	0.003	3.0315 (1.1812 to 7.7803)
Serum alpha-fetoprotein (>90th percentile)	13	14	0.546	0.7016 (0.316 to 1.5576)
Cervicovaginal IL-6 (>90th percentile)	17	12	0.312	1.487 (0.6818 to 3.2454)
Cervicovaginal IGFBP (positive)	3	6	1.0	1.2 (0.2624 to 5.4874)

**Table 4:** Showing sensitivity, specificity, and positive and negative likelihood ratios (LR) of some markers.

Marker	Biological sample	Sensitivity (%) (95% CI)	Specificity (%) (95% CI)	Positive LR	Negative LR
phIGFBP-1	Cervicovaginal fluid	2 (0.005–0.062)	97 (0.96–0.98)	90 (0.28–2.8)	100 (0.97–1.02)
ALP	Serum	14 (0.09–0.21)	99 (0.991–0998)	**35.18** (15.21–81.41)	86 (0.80–0.91)
AFP	Serum	8 (0.04–0.14)	90 (0.84–0.94)	93 (0.45–1.92)	100 (0.95–1.05)
GCSF	Serum	12 (0.08–0.19)	99 (0.984–0.994)	**13** (6.96–24.67)	88 (0.82–0.93)
IL6	Serum	8 (0.04–0.14)	99 (0.984–0.994)	**8.96** (4.44–18.10)	92 (0.87–0.96)
Cervical length	—	14 (0.09–0.21)	98 (0.97–0.99)	**9.85** (5.65–17.16)	80 (0.81–0.92)
